# Potential Correlation Between Eczema and Hematological Malignancies Risk: A Systematic Review and Meta-Analysis

**DOI:** 10.3389/fmed.2022.912136

**Published:** 2022-06-29

**Authors:** Zuohui Liang, Jie Liu, Hongxia Jin, Yirong Teng, Shuangyan Xu, Weimin Yan, Yun Zhu

**Affiliations:** ^1^Department of Dermatology and Venereology, The Sixth Affiliated Hospital of Kunming Medical University, Yuxi, China; ^2^Department of Dermatology and Venereology, The Second Affiliated Hospital of Kunming Medical University, Kunming, China; ^3^Department of Intense Care Unit, Ziyang Hospital of Traditional Chinese Medicine, Ziyang, China; ^4^Department of General Medicine, The Sixth Affiliated Hospital of Kunming Medical University, Yuxi, China; ^5^Department of Dermatology and Venereology, The First Affiliated Hospital of Kunming Medical University, Kunming, China

**Keywords:** eczema, hematological malignancies, Hodgkin's lymphoma, lymphocytic leukemia, myelocytic leukemia, risk

## Abstract

**Background:**

Eczema characterized by itch, sleeplessness, and adverse effects on quality of life is associated with a risk of hematological malignancies. However, there is a controversy pertaining to whether this association implies a greater or lesser risk of hematological cancers. We aimed to explore the link between eczema and hematological malignancies risk.

**Methods:**

We systematically searched PubMed and Embase databases from their inception to February 17, 2022. Two reviewers independently screened articles, extracted data and assessed study quality, respectively. The odds ratios and 95% confidence intervals (CIs) were pooled by using fixed or random-effects models.

**Results:**

29 studies involving 2,521,574 participants examined the contribution of eczema to hematological malignancies. We found that eczema significantly increased the risk of Hodgkin's lymphoma (1.44; 95% CI, 1.07–1.95), myeloma (1.15; 95% CI, 1.04–1.28), and significantly decreased the risk of lymphocytic leukemia (0.91; 95% CI, 0.84–0.99); however, it is not significantly associated with Non-Hodgkin's lymphoma, and myelocytic leukemia.

**Conclusion:**

Eczema has been shown to be associated with the risk of hematological cancer, this association still needs to be verified in large randomized controlled trials.

**Systematic Review Registration:**

https://inplasy.com/, INPLASY202260097.

## Introduction

Hematological malignancies, mainly including lymphoma, leukemia, myeloma, are a common group of highly heterogeneous disorders characterized by uncontrolled proliferation and differentiation of hematopoietic cells (1). In accordance with American Cancer Society, approximately 184,130 new cases and 57,810 deaths of hematological malignancies were estimated in the United States in 2022 (2). Although many researchers have focused on the pathogenesis of hematologic malignancies in recent years and new treatments are available, the long-term survival rate is still unsatisfactory (3–5). Therefore, there is an urgent need to find new specific and non-invasive indicators to evaluate the prognosis of the disease so as to carry out early intervention.

Eczema (atopic dermatitis), one of the most common chronic skin diseases, affecting more than 2.5% of adults and 10% of children (6), may be associated with risk of hematological malignancies (7). However, until now, the role of eczema in the incidence of hematological tumors has been controversial, so this study was designed to explore the relationship between eczema and the risk of hematological cancers.

## Methods

### Search Strategy

According to the *Preferred Reporting Items for Systematic Reviews and Meta-Analyses (PRISMA)* guideline (8, 9), we performed this study. PubMed and Embase databases were systematically searched till February 17, 2022 to find studies performed on the relationship between eczema and hematological cancers. Electronic search strategy is available in Supplementary Material 1. The bibliographies of these literatures were conducted by meticulous analysis of the references listed in the selected articles. Two reviewers (LJ and ZY) did the screening independently, and resolved the conflict through discussion. Firstly, we screened relevant articles based on the titles and abstracts. Then, all papers passing the initial screening would be reviewed the full text.

### Selection Criteria

The inclusion criteria were as follows: all cohort and case-control studies focusing on the relationship between eczema and hematological malignancy; odds ratios (ORs) and 95% confidence intervals (CIs) were provided or calculated. Animal studies, reports with no indication of the association between eczema and cancers, and reviews were excluded. If there is duplication of data in these studies, we selected the study including the largest sample size. The quality of the studies were evaluated according to the Newcastle-Ottawa Scale (NOS) (10). Studies with NOS scores ≥ 7 were considered to be qualified.

### Data Analysis

Two teams (LZH and XSY formed one team; ZY, and LJ formed the other) extracted independently all data. When one study included more than one cohort, we pooled the cohorts and considered each cohort as an independent study. For each independent study, we recorded the following information: first author's name, publication year, study region, study design, type of cancer, participants' sex and age, sample size, and adjustment factors.

The ORs and 95% CIs reported in the studies were pooled by meta-analysis. The Cochrane Q and I2 statistics were used to evaluate heterogeneity (11). When *P* value was <0.10 or the I2 value was >50%, the data were considered heterogeneous, and a random-effects model (12) was applied. Otherwise, a fixed-effects model (13) was used. To further explore the origin of heterogeneity, we performed subgroup analyses by region, study design, and cancer type. To assess the credibility of our results, sensitivity analyses were conducted by excluding each study in turn to estimate the influence of each individual study on the pooled results. Begg's test (14) and Egger's test (15) were used to assess the potential publication bias. STATA software v12.0 (College Station, TX, USA) was used to analyze the data.

## Results

### Study Selection and Basic Characteristics of the Included Studies

**Figure 1:**
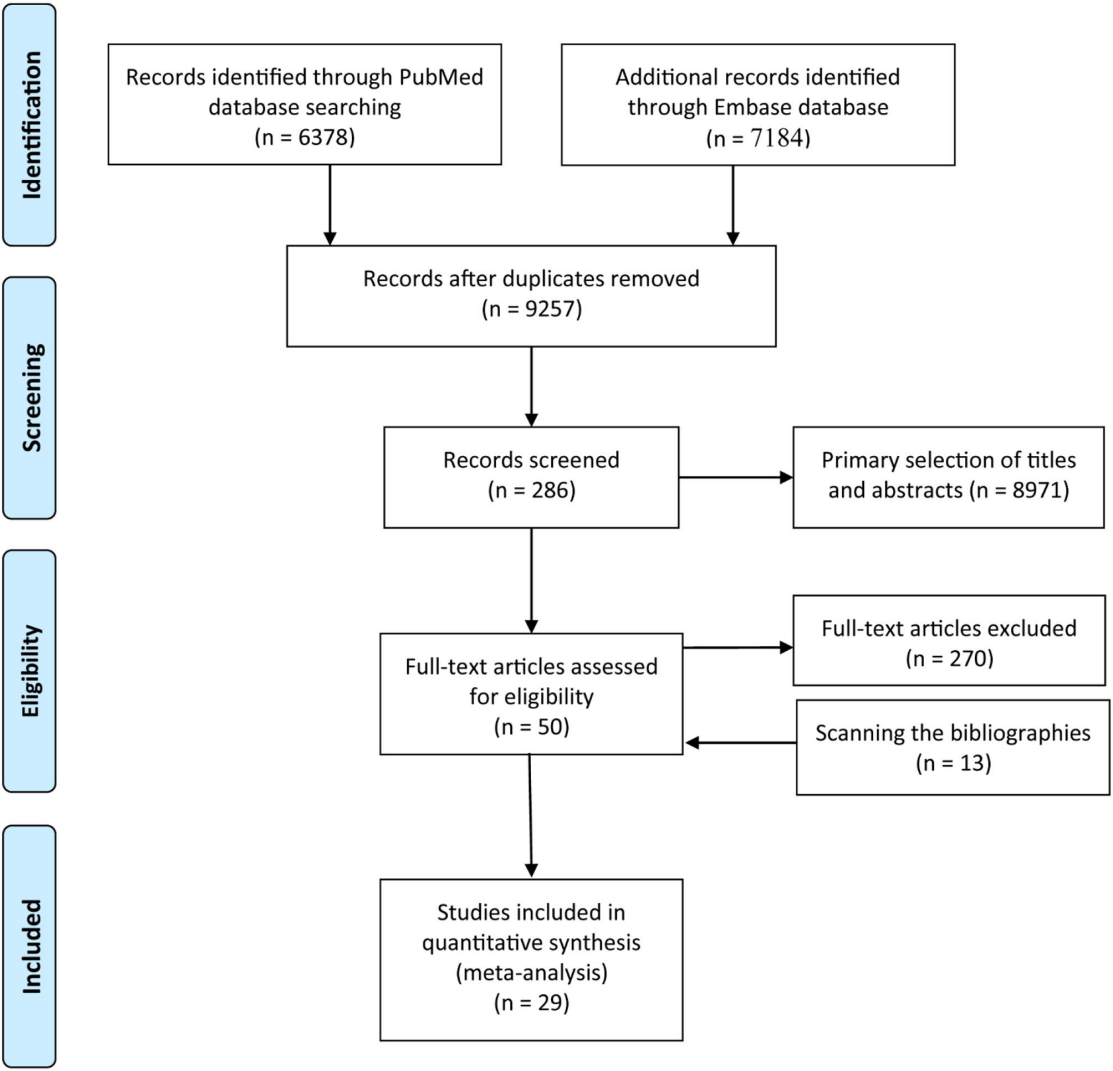
Flow diagram summarizing the pooled analysis phases (i.e., identification, screening, eligibility assessment, and ultimate inclusion).

A total of 13,562 studies were retrieved from the PubMed and the Embase databases, and after removing 4,305 duplicates and further excluding 8,971 studies following title and abstract screening and 270 based on the full article, 16 studies remained. However, 13 additional eligible studies were identified following screening of the bibliographies of relevant studies. Finally, 29 studies involving 2,521,574 participants examined the contribution of eczema to hematological malignancies (Figure 1). Details on the characteristics of the studies are summarized in Supplementary Table 1.

### The Association of Eczema to Non-Hodgkin's Lymphoma

**Figure 2:**
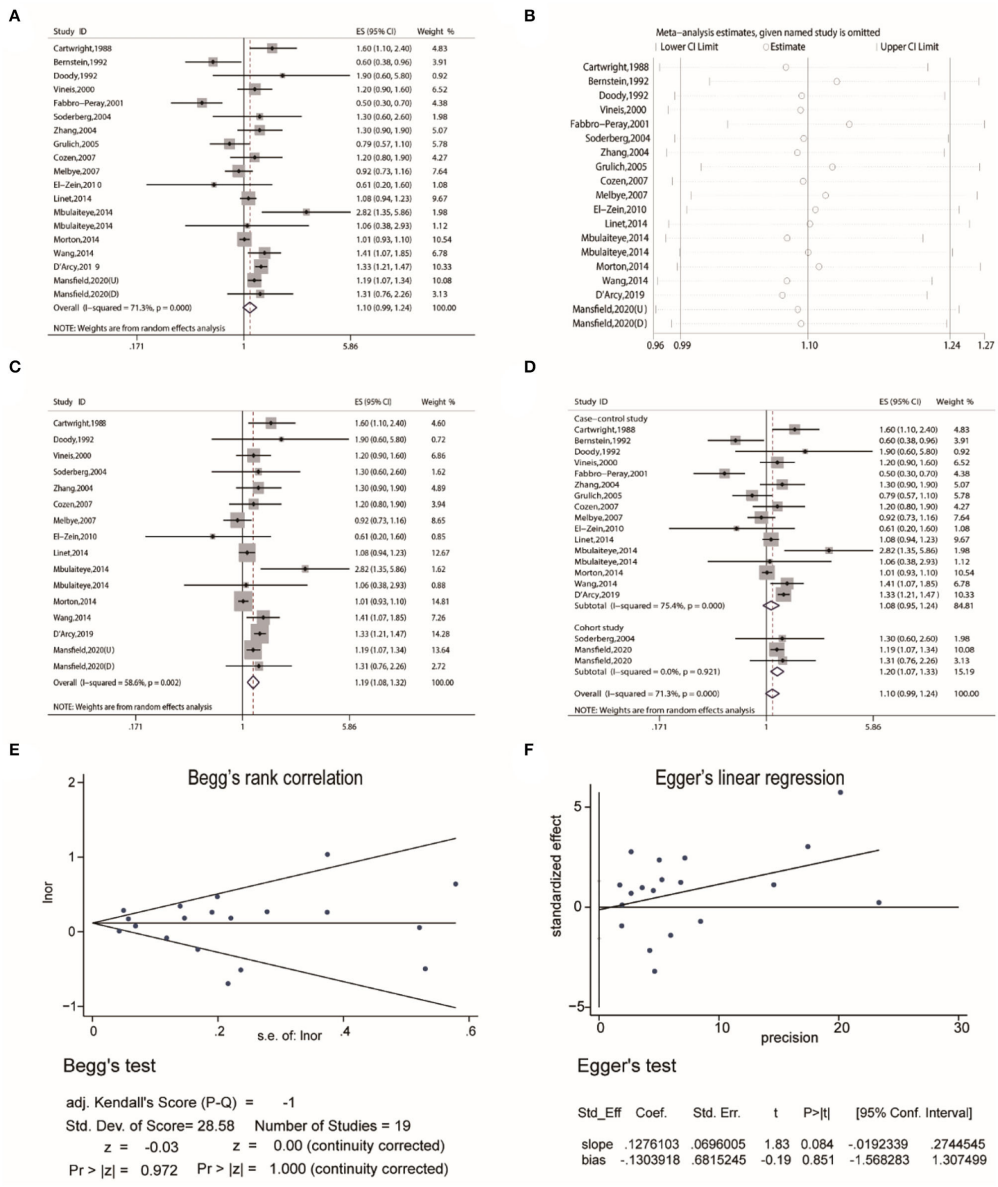
The association between eczema and non-Hodgkin's lymphoma risk. **(A)** Forest plot of the estimated effects of eczema on non-Hodgkin's lymphoma risk. **(B)** Sensitivity analysis conducted by recalculating the pooled results of the primary analysis following the exclusion of one study per iteration. **(C)** Sensitivity analysis conducted by recalculating the pooled results of the primary analysis following the exclusion of the Bernstein et al., Fabbro-Peray et al., and Grulich et al. studies. **(D)** Subgroup analysis of the estimated effects of eczema on non-Hodgkin's lymphoma risk by study design. **(E)** Begg's test indicating the lack of publication bias among such studies. **(F)** Egger's test indicating the lack of publication bias among such studies.

Firstly, the pooled analysis of 17 studies (16–32) indicated that eczema was not significantly associated with an increased risk of Non-Hodgkin's lymphoma (OR, 1.10 [95% CI, 0.99–1.24], P_(ES)_= 0.087); besides, substantial heterogeneity was observed (P_heterogeneity_ = 0.000, I^2^ = 71.3%) (Figure 2A). Sensitivity analysis revealed that after excluding the studies by Bernstein and Ross (18), Fabbro-Peray et al. (21), and Grulich et al. (24). In turn, the overall combined results were altered (Figure 2B), and after excluding the three studies, the resulting data showed that eczema was statistically associated with an increased risk of Non-Hodgkin's lymphoma (OR, 1.19 [95% CI, 1.08–1.32], P_(ES)_ = 0.000; P_heterogeneity_ = 0.002, I^2^ = 58.6%) (Figure 2C). Subgroup pooled analyses were performed according to study design, and we found that eczema was statistically associated with an increased risk of Non-Hodgkin's lymphoma in the cohort subgroup (OR, 1.20 [95% CI, 1.07–1.33], P_(ES)_ = 0.001; P_heterogeneity_ = 0.921, I^2^ = 0.0%), but not the case-control subgroup (OR, 1.08 [95% CI, 0.95–1.24], P_(ES)_ = 0.252; P_heterogeneity_ = 0.000, I^2^ = 75.4%) (Figure 2D). Publication bias tests were performed following Begg's rank correlation and Egger's linear regression tests, which indicated that no publication bias existed among the studies (Begg's: P > |z| = 1.000; Egger's: *P* = 0.851, [95% CI: −1.568–1.307]) (Figures 2E,F).

### The Association of Eczema to Hodgkin's Lymphoma

**Figure 3:**
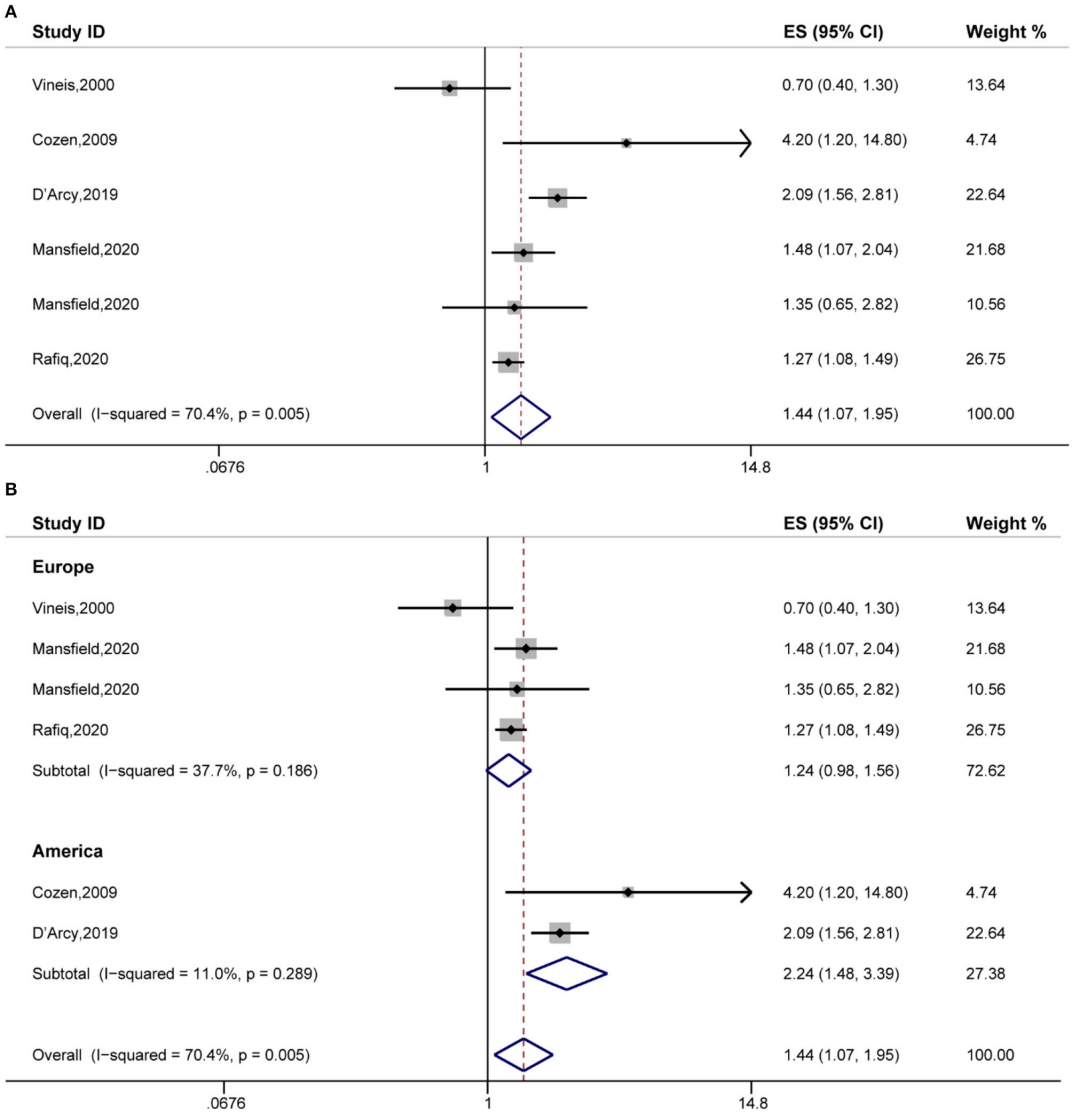
The association between eczema and Hodgkin's lymphoma risk. **(A)** Forest plot of the estimated effects of eczema on Hodgkin's lymphoma risk. **(B)** Subgroup analysis of the estimated effects of eczema on Hodgkin's lymphoma risk.

Secondly, the pooled analysis of six studies (16, 20, 25, 32, 33) indicated that eczema significantly increased the risk of Hodgkin's lymphoma (OR, 1.44 [95% CI, 1.07–1.95], P_(ES)_ = 0.016; P_heterogeneity_ = 0.005, I^2^ = 70.4%) (Figure 3A). Further subgroup analyses performed by study region indicated that eczema significantly increased the risk of Hodgkin's lymphoma in the American subgroup (OR, 2.24 [95% CI, 1.48–3.39], P_(ES)_ = 0.000; P_heterogeneity_ = 0.289, I^2^ = 11.0%) (Figure 3B).

### The Association of Eczema to Lymphocytic Leukemia

**Figure 4:**
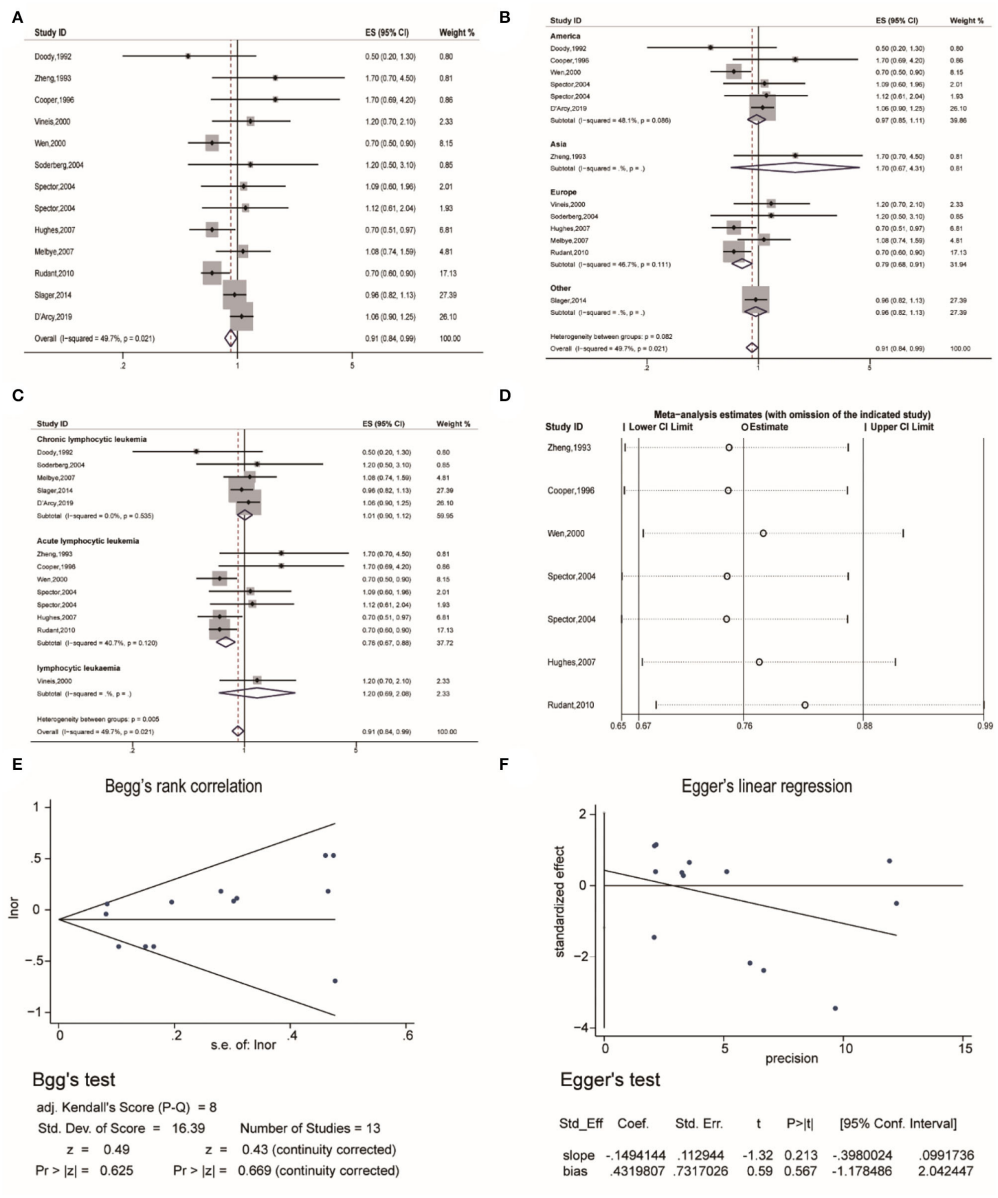
The association between eczema and lymphocytic leukemia risk. **(A)** Forest plot of the estimated effects of eczema on lymphocytic leukemia risk. **(B)** Forest plot for the subgroup analysis by region. **(C)** Forest plot for the subgroup analysis by cancer type. **(D)** Sensitivity analysis regarding the association between eczema and acute lymphocytic leukemia risk. **(E)** Begg's test indicating the lack of publication bias among such studies. **(F)** Egger's test indicating the lack of publication bias among such studies.

Thirdly, the pooled analysis of 13 studies (19, 20, 22, 26, 32, 34–40) indicated that eczema was significantly associated with a decreased risk of lymphocytic leukemia (OR, 0.91 [95% CI, 0.84–0.99], P_(ES)_ = 0.029; P_heterogeneity_ = 0.021, I^2^ = 49.7%) (Figure 4A). Analyses performed in Europe, indicated that eczema significantly decreased the risk of lymphocytic leukemia (OR, 0.79 [95% CI, 0.68–0.91], P_(ES)_ = 0.002; P_heterogeneity_ = 0.111, I^2^ = 46.7%) (Figure 4B). Further subgroup analyses performed by specific cancer type indicated that eczema significantly decreased the risk of acute lymphocytic leukemia (OR, 0.76 [95% CI, 0.67–0.88], P_(ES)_ = 0.000; P_heterogeneity_ = 0.120, I^2^ = 40.7%) (Figure 4C). Sensitivity analysis revealed that after excluding each study in turn, the overall combined results were not altered (Figure 4D). Publication bias tests were performed following Begg's rank correlation and Egger's linear regression tests, which indicated that no publication bias existed among the studies (Begg's: P > |z| = 0.669; Egger's: *P* = 0·567, [95% CI:−1.178–2.042]) (Figures 4E,F).

### The Association of Eczema to Myelocytic Leukemia

**Figure 5:**
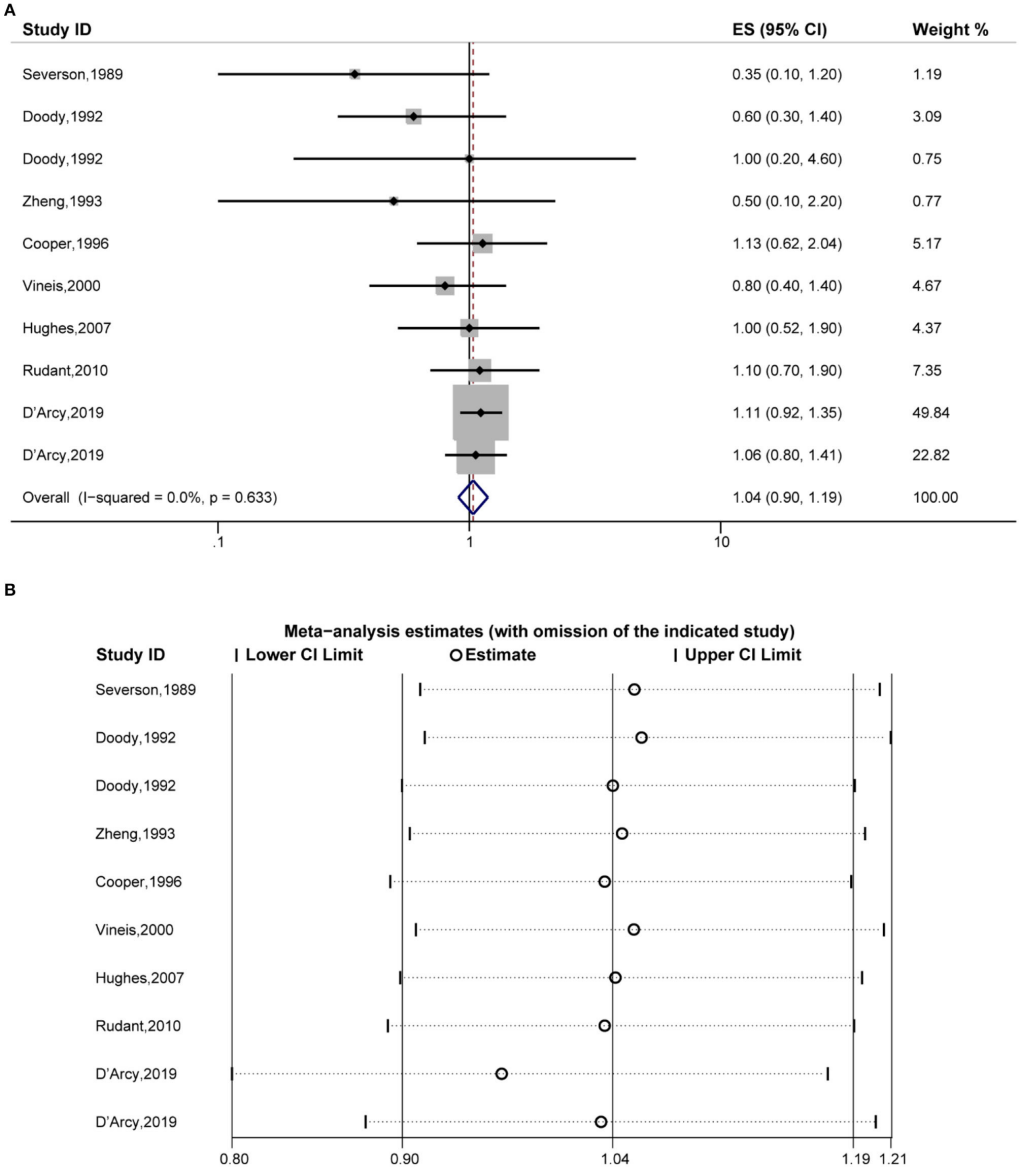
Forest plot of the estimated effects of eczema on myelocytic leukemia risk and the sensitivity analysis. **(A)** Forest plot of eczema on myelocytic leukemia risk. **(B)** Sensitivity analysis was conducted by recalculating the pooled results of the primary analysis following the exclusion of one study per iteration.

Fourthly, the pooled analysis of eight studies (19, 20, 32, 34, 35, 38, 39, 41) indicated that eczema was not significantly associated with an increased risk of myelocytic leukemia (OR, 1.04 [95% CI, 0.90–1.19], P_(ES)_ = 0.616; P_heterogeneity_ = 0.633, I^2^ = 0.0%) (Figure 5A), and the sensitivity analysis revealed that excluding each study in turn did not alter the overall combined results (Figure 5B).

### The Association of Eczema to Myeloma

**Figure 6:**
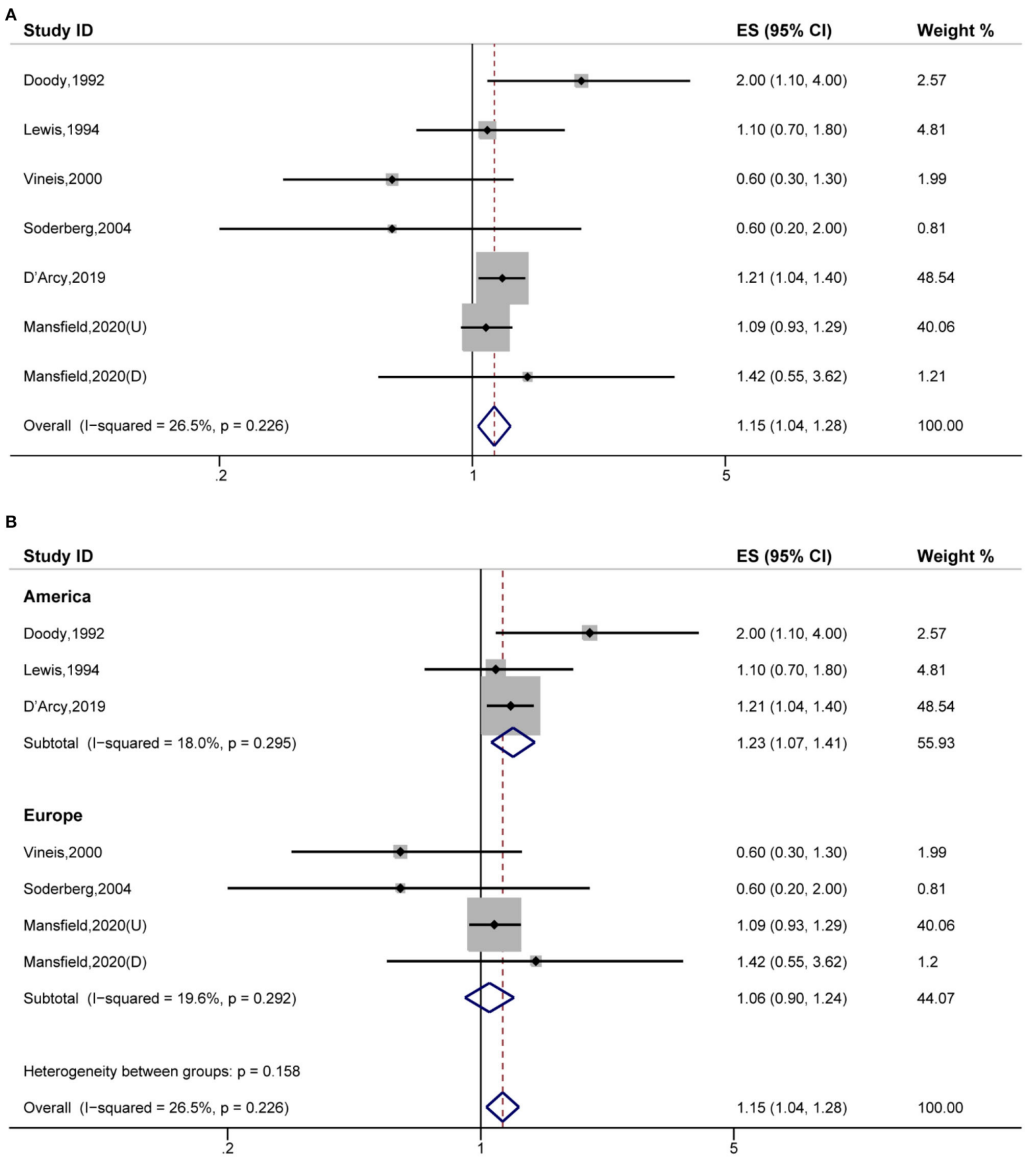
Forest plot of the estimated effects of eczema on myeloma risk. **(A)** Forest plot. **(B)** Forest plot for the subgroup analysis by region.

Fifthly, the pooled analysis of six studies (16, 19, 20, 22, 32, 42) indicated that eczema was significantly associated with an increased risk of myeloma (OR, 1.15 [95% CI, 1.04–1.28], P_(ES)_ = 0.008; P_heterogeneity_ = 0.226, I^2^ = 26.5%) (Figure 6A). Further analyses performed in America indicated that eczema significantly increased the risk of myeloma (OR, 1.23 [95% CI, 1.07–1.41], P_(ES)_ = 0.004; P_heterogeneity_ = 0.295, I^2^ = 18.0%) (Figure 6B).

## Discussion

Eczema characterized by itch, sleeplessness, and adverse effects on quality of life is associated with a risk of several diseases (43). A meta-analysis found that eczema was associated with increased risk of various cardiovascular diseases in cohort studies (44). A recent study also suggested that eczema is associated with an increased risk of developing depression and anxiety (45). But at present, the relationship between eczema and hematological tumors is still controversial, and our study aims to resolve this issue.

Our study indicated that the relationship between eczema and the risk of various types of hematological tumors was different. Firstly, our study showed no statistical relationship between eczema and an increased risk of non-Hodgkin's lymphoma, but the new results after excluding three articles with visible bias in sensitivity analysis showed that eczema was associated with an increased risk of non-Hodgkin's lymphoma, which was consistent with the findings of the cohort subgroup. In addition, eczema was significantly associated with the increased risk of Hodgkin's lymphoma and myeloma. Eczema was likely to the increased risk of myelocytic leukemia without statistical significance. On the contrary, eczema was statistically related with the decreased risk of lymphocytic leukemia. This difference is possibly due to the different risk factor spectrum of non-Hodgkin's lymphoma, Hodgkin's lymphoma and other hematological tumors (46). Of course, the existence of confounding bias and different treatment detections (especially immunomodulatory systemic therapeutics) may also be important reasons (16). What's more, T-cell NHL presenting in an indolent manner is always misdiagnosed as a non-neoplastic skin condition, and this diagnostic confusion could slightly attenuate the link between eczema and NHL risk (32).

Further subgroup analyses suggested that different study regions affect the association between eczema and hematological malignancies. Eczema was statistically associated with the increased risk of Hodgkin's lymphoma and myeloma in the American subgroup, but this link would be substantially attenuated in the European subgroup. This result was resulted from differences in race and environmental factors (47, 48).

In addition, eczema was significantly associated with the decreased risk of acute lymphocytic leukemia, but it was not statistically associated with the risk of chronic lymphocytic leukemia. This may be because some treatments for eczema (Glucocorticoids, biological inhibitors, etc.) may have a certain preventive effect on acute lymphoid leukemia (49), but this effect is not obvious for chronic lymphoid leukemia. Meanwhile, patients with eczema often receive phototherapy, which may affect their risk of cancer because of its therapeutic effect on cancer (50).

Although our study addresses the shortcomings of a single study with a small sample size and a non-universal population investigated, there are still many limitations. Firstly, there is a different degree of heterogeneity in some of our results, which affects the reliability of our results, but we performed subgroup analyses to look for sources of heterogeneity; In addition, although most of the studies we included had adjusted for confounding factors, the confounding factors adjusted were different.

## Conclusion

This study demonstrated that eczema significantly increases the risk of Hodgkin's lymphoma, myeloma, and significantly decreases the risk of lymphocytic leukemia. Although eczema has been shown to be associated with the risk of many hematological malignancies, this association still needs to be verified in large randomized controlled trials. At the same time, the mechanism of eczema leading to cancer is needed to explore.

## Data Availability Statement

The original contributions presented in the study are included in the article/Supplementary Material, further inquiries can be directed to the corresponding author/s.

## Author Contributions

Material preparation, data collection, and analysis were performed by ZL, SX, JL, HJ, YT, and YZ. The first draft of the manuscript was written by ZL, JL, and HJ. All authors contributed to the study conception, design, commented on previous versions of the manuscript, and approved the final manuscript.

## Funding

We would like to thank the National Natural Science Foundation of China (grant numbers 81760136 and 31960136), the Special and Joint Program of Yunnan Provincial Science and Technology Department and Kunming Medical University (202001AY070001-097), the Yunnan health training project of high level talents, and the Scientific Research Fund project of Yunnan Education Department (2021J0304 and 2019J1308) for supporting our work.

## Conflict of Interest

The authors declare that the research was conducted in the absence of any commercial or financial relationships that could be construed as a potential conflict of interest.

## Publisher's Note

All claims expressed in this article are solely those of the authors and do not necessarily represent those of their affiliated organizations, or those of the publisher, the editors and the reviewers. Any product that may be evaluated in this article, or claim that may be made by its manufacturer, is not guaranteed or endorsed by the publisher.
