# Supplementary material for: Potential Correlation Between Eczema and Hematological Malignancies Risk: A Systematic Review and Meta-Analysis
Source: Front Med (Lausanne). 2022 Jun 29;9:912136. doi: 10.3389/fmed.2022.912136 (PMC9277696; doi:10.3389/fmed.2022.912136)
Supplement: Supplementary file 1 [file Data_Sheet_1.docx]

Supplementary Material

# Supplementary Data

## Literature search strategies.

Database: PubMed (from inception to February 17, 2022)

Search strategy:

Terms specific to Eczema

#1. "Eczema"[Mesh] OR "Eczema, Dyshidrotic"[Mesh] OR "Dermatitis, Atopic"[Mesh]

#2. (((((((((((((((((((((((((((((((((Eczemas[Text Word]) OR (Dermatitis, Eczematous[Text Word])) OR (Dermatitides, Eczematous[Text Word])) OR (Eczematous Dermatitides[Text Word])) OR (Eczematous Dermatitis[Text Word])) OR (Dyshidrotic Eczema[Text Word])) OR (Dyshidrotic Eczemas[Text Word])) OR (Eczemas, Dyshidrotic[Text Word])) OR (Vesicular Palmoplantar Eczema[Text Word])) OR (Eczema, Vesicular Palmoplantar[Text Word])) OR (Eczemas, Vesicular Palmoplantar[Text Word])) OR (Palmoplantar Eczema, Vesicular[Text Word])) OR (Palmoplantar Eczemas, Vesicular[Text Word])) OR (Vesicular Palmoplantar Eczemas[Text Word])) OR (Pompholyx[Text Word])) OR (Eczema, Dyshydrotic[Text Word])) OR (Dyshydrotic Eczema[Text Word])) OR (Dyshydrotic Eczemas[Text Word])) OR (Eczemas, Dyshydrotic[Text Word])) OR (Atopic Dermatitides[Text Word])) OR (Atopic Dermatitis[Text Word])) OR (Dermatitides, Atopic[Text Word])) OR (Neurodermatitis, Atopic[Text Word])) OR (Atopic Neurodermatitides[Text Word])) OR (Atopic Neurodermatitis[Text Word])) OR (Neurodermatitides, Atopic[Text Word])) OR (Neurodermatitis, Disseminated[Text Word])) OR (Disseminated Neurodermatitides[Text Word])) OR (Disseminated Neurodermatitis[Text Word])) OR Neurodermatitides, Disseminated[Text Word])) OR (Eczema, Atopic[Text Word])) OR (Atopic Eczema[Text Word])) OR (Eczema, Infantile[Text Word])) OR (Infantile Eczema[Text Word])

#3. #1 OR #2

Terms specific to Study Design

#4. ("Cohort Studies"[Mesh]) OR ("Case-Control Studies"[Mesh]) OR ("risk"[Mesh]) OR ((hazard[Text Word] OR odds[Text Word]) AND ratio*[Text Word]) OR (relative[Text Word] AND risk[Text Word])

Combination of terms to identify Eczema

#5. #3 AND #4

#6. #5 AND ("case reports"[pt] OR letter[pt] OR review[pt] OR editorial[pt] OR comment[pt] OR "practice guideline"[pt] OR "historical article"[pt] OR news[pt] OR meta-analysis[pt])

#7. #5 NOT #6

((("Eczema"[Mesh] OR "Eczema, Dyshidrotic"[Mesh] OR "Dermatitis, Atopic"[Mesh]) OR ((((((((((((((((((((((((((((((((((Eczemas[Text Word]) OR (Dermatitis, Eczematous[Text Word])) OR (Dermatitides, Eczematous[Text Word])) OR (Eczematous Dermatitides[Text Word])) OR (Eczematous Dermatitis[Text Word])) OR (Dyshidrotic Eczema[Text Word])) OR (Dyshidrotic Eczemas[Text Word])) OR (Eczemas, Dyshidrotic[Text Word])) OR (Vesicular Palmoplantar Eczema[Text Word])) OR (Eczema, Vesicular Palmoplantar[Text Word])) OR (Eczemas, Vesicular Palmoplantar[Text Word])) OR (Palmoplantar Eczema, Vesicular[Text Word])) OR (Palmoplantar Eczemas, Vesicular[Text Word])) OR (Vesicular Palmoplantar Eczemas[Text Word])) OR (Pompholyx[Text Word])) OR (Eczema, Dyshydrotic[Text Word])) OR (Dyshydrotic Eczema[Text Word])) OR (Dyshydrotic Eczemas[Text Word])) OR (Eczemas, Dyshydrotic[Text Word])) OR (Atopic Dermatitides[Text Word])) OR (Atopic Dermatitis[Text Word])) OR (Dermatitides, Atopic[Text Word])) OR (Neurodermatitis, Atopic[Text Word])) OR (Atopic Neurodermatitides[Text Word])) OR (Atopic Neurodermatitis[Text Word])) OR (Neurodermatitides, Atopic[Text Word])) OR (Neurodermatitis, Disseminated[Text Word])) OR (Disseminated Neurodermatitides[Text Word])) OR (Disseminated Neurodermatitis[Text Word])) OR (Neurodermatitides, Disseminated[Text Word])) OR (Eczema, Atopic[Text Word])) OR (Atopic Eczema[Text Word])) OR (Eczema, Infantile[Text Word])) OR (Infantile Eczema[Text Word]))) AND (("Cohort Studies"[Mesh]) OR ("Case-Control Studies"[Mesh]) OR ("risk"[Mesh]) OR ((hazard[Text Word] OR odds[Text Word]) AND ratio*[Text Word]) OR (relative[Text Word] AND risk[Text Word]))) NOT ((("case reports"[pt] OR letter[pt] OR review[pt] OR editorial[pt] OR comment[pt] OR "practice guideline"[pt] OR "historical article"[pt] OR news[pt] OR meta-analysis[pt])) AND ((("Eczema"[Mesh] OR "Eczema, Dyshidrotic"[Mesh] OR "Dermatitis, Atopic"[Mesh]) OR ((((((((((((((((((((((((((((((((((Eczemas[Text Word]) OR (Dermatitis, Eczematous[Text Word])) OR (Dermatitides, Eczematous[Text Word])) OR (Eczematous Dermatitides[Text Word])) OR (Eczematous Dermatitis[Text Word])) OR (Dyshidrotic Eczema[Text Word])) OR (Dyshidrotic Eczemas[Text Word])) OR (Eczemas, Dyshidrotic[Text Word])) OR (Vesicular Palmoplantar Eczema[Text Word])) OR (Eczema, Vesicular Palmoplantar[Text Word])) OR (Eczemas, Vesicular Palmoplantar[Text Word])) OR (Palmoplantar Eczema, Vesicular[Text Word])) OR (Palmoplantar Eczemas, Vesicular[Text Word])) OR (Vesicular Palmoplantar Eczemas[Text Word])) OR (Pompholyx[Text Word])) OR (Eczema, Dyshydrotic[Text Word])) OR (Dyshydrotic Eczema[Text Word])) OR (Dyshydrotic Eczemas[Text Word])) OR (Eczemas, Dyshydrotic[Text Word])) OR (Atopic Dermatitides[Text Word])) OR (Atopic Dermatitis[Text Word])) OR (Dermatitides, Atopic[Text Word])) OR (Neurodermatitis, Atopic[Text Word])) OR (Atopic Neurodermatitides[Text Word])) OR (Atopic Neurodermatitis[Text Word])) OR (Neurodermatitides, Atopic[Text Word])) OR (Neurodermatitis, Disseminated[Text Word])) OR (Disseminated Neurodermatitides[Text Word])) OR (Disseminated Neurodermatitis[Text Word])) OR (Neurodermatitides, Disseminated[Text Word])) OR (Eczema, Atopic[Text Word])) OR (Atopic Eczema[Text Word])) OR (Eczema, Infantile[Text Word])) OR (Infantile Eczema[Text Word]))) AND (("Cohort Studies"[Mesh]) OR ("Case-Control Studies"[Mesh]) OR ("risk"[Mesh]) OR ((hazard[Text Word] OR odds[Text Word]) AND ratio*[Text Word]) OR (relative[Text Word] AND risk[Text Word]))))

**Database: Embase (from inception to February 17, 2022)**

Search strategy:

Terms specific to Eczema

#1. 'eczema'/exp OR 'eczema':ti,ab

#2. 'atopic eczema'/exp OR 'atopic eczema':ti,ab

#3. 'dyshidrotic eczema'/exp OR 'dyshidrotic eczema':ti,ab

#4. 'atopic dermatitis'/exp OR 'atopic dermatitis':ti,ab

#5. #1 OR #2 OR #3 OR #4

Terms specific to Study Design

#6. 'cohort study'/exp OR (cohort stud*):ti,ab

#7. 'case-control study'/exp OR 'case-control stud*':ti,ab

#8. 'risk'/exp OR risk:ti,ab OR 'ratio'/exp OR ratio:ti,ab

#9. #6 OR #7 OR #8

Combination of terms to identify Eczema

#10. #5 AND #9

#11. #10 AND ([article]/lim OR [article in press]/lim) AND [humans]/lim AND [clinical study]/lim AND [embase]/lim
