# Supplementary material for: Potential Correlation Between Eczema and Hematological Malignancies Risk: A Systematic Review and Meta-Analysis
Source: Front Med (Lausanne). 2022 Jun 29;9:912136. doi: 10.3389/fmed.2022.912136 (PMC9277696; doi:10.3389/fmed.2022.912136)
Supplement: Supplementary file 2 [file Table_1.docx]

Table1. The base characteristics of the included literature

| Study,year | Study design | Region | Cancer | Sample | Case | Control | Age | Sex | Adjustments | Statistical analysis | NOS-score |
| --- | --- | --- | --- | --- | --- | --- | --- | --- | --- | --- | --- |
| Bernard,1984 | Case-control | Europe | Lymphoid maligancy | 570 | 285 | 285 | ≥15 | Female and male | Age, sex and geographic area | Mantel-Haenszel and Miettinen | 8 |
| Cartwright,1988 | Case-control | Europe | Non-Hodgkin lymphoma | 1161 | 437 | 724 | ≥15 | Female and male | Age and sex | Mantel-Haenszel and Miettinen | 5 |
| Severson,1989 | Case-control | America | Acute myelocytic leukemia | 231 | 98 | 133 | 20-79 | Female and male | Allergy，Drugs，Allergens，Other allergies | Mantel-Haenszel and Miettinen | 6 |
| Bernstein,1992 | Case-control | America | Non-Hodgkin lymphoma | 619 | NA | NA | 19-75 | Female and male | NA | Conditional logistic regression | 6 |
| Doody,1992 | Case-control | America | Leukemia | 676 | 299 | 377 | ≥15 | Female and male | Region,sex,age,number of years as a member in the program,and calendar year in which membership began | Conditional logistic regression | 6 |
| Doody,1992 | Case-control | America | Multiple myeloma | 394 | 175 | 219 | ≥15 | Female and male | Region,sex,age,number of years as a member in the program,and calendar year in which membership began | Conditional logistic regression | 6 |
| Doody,1992 | Case-control | America | Non-Hodgkin lymphoma | 291 | 100 | 191 | ≥15 | Female and male | Region,sex,age,number of years as a member in the program,and calendar year in which membership began | Conditional logistic regression |  |
| Zheng,1993 | Case-control | Asia | Acute lymphocytic leukemia | 583 | 81 | 502 | ≥15 | Female and male | Sex, age, income, and occupational exposures | Mantel- Haenszel stratified estimation method and multiple logistic regression | 8 |
| Zheng,1993 | Case-control | Asia | Acute non-lymphocyticleukemia (ANLL) | 738 | 236 | 502 | ≥15 | Female and male | Sex, age, income, and occupational exposures | Mantel- Haenszel stratified estimation method and multiple logistic regression | 8 |
| Zheng,1993 | Case-control | Asia | Chronic myeloidleukemia (CML) | 581 | 79 | 502 | ≥15 | Female and male | Sex, age, income, and occupational exposures | Mantel- Haenszel stratified estimation method and multiple logistic regression | 8 |
| Lewis,1994 | Case-control | America | Multiple myeloma | 2704 | 25 | 82 | 30-79 | Female and male | Medical conditions,biologic and immunologic grouping by race | Unconditional logistic regression | 7 |
| Cooper,1996 | Case-control | America | Acute myelocytic leukemia | 1251 | 27 | 637 | 18-79 | Female and male | Age,gender,race,education，and smoking | Logistic regression | 8 |
| Cooper,1996 | Case-control | America | Acute lymphocytic leukemia | 760 | 9 | 637 | 18-79 | Female and male | Age,gender,race,education，and smoking | Logistic regression |  |
| Vineis,2000 | Case-control | Europe | Non-Hodgkin lymphoma | 3,106 | 1388 | 1,718 | 20-74 | Female and male | Age and gender | Stratified (Mantel-Haenszel) and multivariate procedures (logistic regression) | 8 |
| Vineis,2000 | Case-control | Europe | Hodgkin’s Lymphoma | 2,072 | 354 | 1,718 | 20-74 | Female and male | Age and gender | Stratified (Mantel-Haenszel) and multivariate procedures (logistic regression) | 8 |
| Vineis,2000 | Case-control | Europe | multiple myeloma | 1,981 | 263 | 1,718 | 20-74 | Female and male | Age and gender | Stratified (Mantel-Haenszel) and multivariate procedures (logistic regression) | 8 |
| Vineis,2000 | Case-control | Europe | lymphocytic leukaemia | 1,979 | 261 | 1,718 | 20-74 | Female and male | Age and gender | Stratified (Mantel-Haenszel) and multivariate procedures (logistic regression) | 8 |
| Vineis,2000 | Case-control | Europe | myeloid leukaemia | 2,031 | 313 | 1,718 | 20-74 | Female and male | Age and gender | Stratified (Mantel-Haenszel) and multivariate procedures (logistic regression) | 8 |
| Wen,2000 | Case-control | America | Acute lymphocytic leukemia | 3828 | 1842 | 1986 | ≤15 | Female and male | number of siblings, breastfeeding, maternal education, race, and family income | Conditional logistic regression model | 8 |
| Fabbro-Peray,2001 | Case-control | Europe | Non-Hodgkin lymphoma | 1,470 | 445 | 1,025 | ≥18 | Female and male | Age, Sex, Level of education, Setting and Reported medical history, | Mantel Haenszel method | 7 |
| Soderberg,2004 | Cohort | Europe | Leukemia | 16,539 | NA | NA | NA | Female and male | age and sex | Cox Proportional Hazards Regression Model | 9 |
| Soderberg,2004 | Cohort | Europe | Chronic lymphocytic leukemia | 16,539 | NA | NA | NA | Female and male | Age and sex | Cox Proportional Hazards Regression Model | 9 |
| Soderberg,2004 | Cohort | Europe | Myeloma | 16,539 | NA | NA | NA | Female and male | Age and sex | Cox Proportional Hazards Regression Model | 9 |
| Soderberg,2004 | Cohort | Europe | Non-Hodgkin lymphoma | 16,539 | NA | NA | NA | Female and male | Age and sex | Cox Proportional Hazards Regression Model | 9 |
| Spector,2004 | Case-control | America | Acute lymphocytic leukemia | 898 | 180 | 718 | 1.5-6 | Female and male | Age, gender, race and HMO | Conditional logistic regression | 8 |
| Spector,2004 | Case-control | America | Acute lymphoblastic leukaemia | 733 | 147 | 586 | 1.5-6 | Female and male | Age, gender, race and HMO | Conditional logistic regression | 8 |
| Zhang,2004 | Case-control | America | Non-Hodgkin lymphoma | 1,318 | 601 | 717 | 21-84 | Female | Age, BMI, menopausal status, and family history | Unconditional logistic regression | 9 |
| Grulich,2005 | Case-control | Australia | Non-Hodgkin lymphoma | 1,217 | 704 | 694 | during lifetime | Female and male | Matching variables (age as a continuous variable, sex, and state) and ethnicity | Unconditional logistic regression | 9 |
| Cozen,2007 | Case-control | America | Non-Hodgkin lymphoma | 2,378 | 1,321 | 1,057 | 20-74 | Female and male | Study center, age, gender, race, birth order and education | Multiple logistic regression | 7 |
| Cozen,2007 | Case-control | America | Diffuse large B-cell lymphoma | 2,378 | 1,321 | 1,057 | 20-74 | Female and male | Study center, age, gender, race, birth order and education | Multiple logistic regression | 7 |
| Cozen,2007 | Case-control | America | Follicular lymphoma | 2,378 | 1,321 | 1,057 | 20-74 | Female and male | Study center, age, gender, race, birth order and education | Multiple logistic regression | 7 |
| Hughes,2007 | Case-control | Europe | All leukaemias combined | 2176 | 839 | 1337 | ≤14 | Female and male | Sex, age (in single years), region and deprivation index.– | Unconditional logistic regression models | 8 |
| Hughes,2007 | Case-control | Europe | Acute lymphocytic leukemia | 2057 | 720 | 1337 | ≤14 | Female and male | Sex, age (in single years), region and deprivation index.– | Unconditional logistic regression models | 8 |
| Hughes,2007 | Case-control | Europe | common-ALL/precursor B-cell ALL | 1875 | 538 | 1337 | ≤14 | Female and male | Sex, age, region and deprivation index. | Unconditional logistic regression models | 8 |
| Hughes,2007 | Case-control | Europe | Acute myelocytic leukemia | 1438 | 101 | 1337 | ≤14 | Female and male | Sex, age, region and deprivation index. | Unconditional logistic regression models | 8 |
| Melbye,2007 | Case-control | Europe | Non-Hodgkin lymphoma | 6144 | 3007 | 3137 | 18-74 | Female and male | Aage, sex, country, birth order, education, and outdoor occupation. | Logistic regression | 7 |
| Melbye,2007 | Case-control | Europe | Chronic lymphocytic leukemia | 3879 | 742 | 3137 | 18-74 | Female and male | Aage, sex, country, birth order, education, and outdoor occupation. | Logistic regression | 7 |
| Cozen,2009 | Case-control | America | Hodgkin’s Lymphoma | 188 | NA | NA | <51 | Female and male | Appendectomy, smoked cigarettes, ever/never, relatively more behaviors resulting in early oral exposures | Multivariable conditional logistic regression | 6 |
| El-Zein,2010 | Case-control | America | Non-Hodgkin lymphoma | 709 | 197 | 512 | 35-70 | Males | Age, income, respondent status, ancestry, birthplace, and smoking | Logistic regression | 6 |
| Rudant,2010 | Case-control | Europe | Acute lymphocytic leukemia | 2128 | 634 | 1494 | 1-14 | Female and male | Stratification variables, age, gender, parental professional category, and degree of urbanization | Unconditional logistic regression | 8 |
| Rudant,2010 | Case-control | Europe | Acute myelocytic leukemia | 1580 | 86 | 1494 | 1-14 | Female and male | Stratification variables, age, gender, parental professional category, and degree of urbanization | Unconditional logistic regression | 8 |
| Linet,2014 | Case-control | Other | Follicular lymphoma | 19141 | 2770 | 16371 | 16-98 | Female and male | Age, race/ethnicity, sex | Logistic regression models | 8 |
| Mbulaiteye,2014 | Case-control | Other | Burkitt Lymphoma or Burkitt-like lymphoma | 3859 | 100 | 3759 | ＜50 | Female and male | Study, age, sex, and race/ethnicity | Fixed-effects logistic regression models | 7 |
| Mbulaiteye,2014 | Case-control | Other | Burkitt Lymphoma or Burkitt-like lymphoma | 10396 | 126 | 10270 | ≥50 | Female and male | Study, age, sex, and race/ethnicity | Fixed-effects logistic regression models | 7 |
| Morton,2014 | Case-control | Other | Non-Hodgkin lymphoma | 3975 | 1712 | 2263 | median age 41 year | Female and male | Age, race/ethnicity,sex, and study. | Unconditional fixed effects logistic regression | 7 |
| Slager,2014 | Case-control | Other | Chronic lymphocytic leukemia | 16172 | 2260 | 13912 | 64(28-93)/60(17-97) | Female and male | Age, sex, race/ethnicity, and study | Multivariate logistic regression analyses | 7 |
| Wang,2014 | Case-control | Other | Non-Hodgkin lymphoma(T cell lymphomas) | 15188 | 537 | 14651 | NA | Female and male | Age, race, sex, study, and all other variables in the model | Logistic regression | 7 |
| D'Arcy,2019 | Case-control | America | Hodgkin’s Lymphoma | ###### | 3,054 | ###### | 66-99 | Female and male | Sex, age, race, calendar year of selection, and measures of socioeconomic status and healthcare utilization | Logistic regression analysis | 7 |
| D'Arcy,2019 | Case-control | America | Non-Hodgkin lymphoma | ###### | 91,381 | ###### | 66-99 | Female and male | Sex, age, race, calendar year of selection, and measures of socioeconomic status and healthcare utilization | Logistic regression analysis | 7 |
| D'Arcy,2019 | Case-control | America | Chronic lymphocytic leukemia | ###### | 25,107 | ###### | 66-99 | Female and male | Sex, age, race, calendar year of selection, and measures of socioeconomic status and healthcare utilization | Logistic regression analysis | 7 |
| D'Arcy,2019 | Case-control | America | Myeloma | ###### | 26,107 | ###### | 66-99 | Female and male | Sex, age, race, calendar year of selection, and measures of socioeconomic status and healthcare utilization | Logistic regression analysis | 7 |
| D'Arcy,2019 | Case-control | America | Acute myelocytic leukemia | ###### | 13,031 | ###### | 66-99 | Female and male | Sex, age, race, calendar year of selection, and measures of socioeconomic status and healthcare utilization | Logistic regression analysis | 7 |
| D'Arcy,2019 | Case-control | America | Chronic myeloidleukemia (CML) | ###### | 5,795 | ###### | 66-99 | Female and male | Sex, age, race, calendar year of selection, and measures of socioeconomic status and healthcare utilization | Logistic regression analysis | 7 |
| Mansfield,2020 | Cohort | Europe | Non-Hodgkin lymphoma | ###### | NA | NA | 24.9-60.7 | Female and male | Sex, primary care practice and date and age | Cox Proportional Hazards Regression Model | 8 |
| Mansfield,2020 | Cohort | Europe | Hodgkin’s Lymphoma | ###### | NA | NA | 24.9-60.7 | Female and male | Sex, primary care practice and date and age | Cox Proportional Hazards Regression Model | 8 |
| Mansfield,2020 | Cohort | Europe | Leukemia | ###### | NA | NA | 24.9-60.7 | Female and male | Sex, primary care practice and date and age | Cox Proportional Hazards Regression Model | 8 |
| Mansfield,2020 | Cohort | Europe | Multiple myeloma | ###### | NA | NA | 24.9-60.7 | Female and male | Sex, primary care practice and date and age | Cox Proportional Hazards Regression Model | 8 |
| Mansfield,2020 | Cohort | Europe | Non-Hodgkin lymphoma | ###### | NA | NA | 1.7-20.8 | Female and male | Sex,date and age | Cox Proportional Hazards Regression Model | 8 |
| Mansfield,2020 | Cohort | Europe | Hodgkin’s Lymphoma | ###### | NA | NA | 1.7-20.8 | Female and male | Sex,date and age | Cox Proportional Hazards Regression Model | 8 |
| Mansfield,2020 | Cohort | Europe | Leukemia | ###### | NA | NA | 1.7-20.8 | Female and male | Sex,date and age | Cox Proportional Hazards Regression Model | 8 |
| Mansfield,2020 | Cohort | Europe | Multiple myeloma | ###### | NA | NA | 1.7-20.8 | Female and male | Sex,date and age | Cox Proportional Hazards Regression Model | 8 |
| Rafiq,2020 | Case-control | Europe | Hodgkin’s Lymphoma | 8652 | 1236 | 7416 | ≤50 | Female and male | Age, sex and follow-up time and adjusted for other variables in the model | Multivariable conditional  logistic regression | 8 |
